# Generalizable deep temporal models for predicting episodes of sudden hypotension in critically ill patients: a personalized approach

**DOI:** 10.1038/s41598-020-67952-0

**Published:** 2020-07-10

**Authors:** Brandon Chan, Brian Chen, Alireza Sedghi, Philip Laird, David Maslove, Parvin Mousavi

**Affiliations:** 1 School of Computing, Queen’s University, Kingston, K7L 3N6 Canada; 2 Department of Critical Care Medicine, Queen’s University, Kingston, K7L 2V7 Canada

**Keywords:** Information technology, Biomedical engineering, Medical research, Cardiology, Biomarkers

## Abstract

The vast quantities of data generated and collected in the Intensive Care Unit (ICU) have given rise to large retrospective datasets that are frequently used for observational studies. The temporal nature and fine granularity of much of the data collected in the ICU enable the pursuit of predictive modeling. In particular, forecasting acute hypotensive episodes (AHE) in intensive care patients has been of interest to researchers in critical care medicine. Given an advance warning of an AHE, care providers may be prompted to search for evolving disease processes and help mitigate negative clinical outcomes. However, the conventionally adopted definition of an AHE does not account for inter-patient variability and is restrictive. To reflect the wider trend of global clinical and research efforts in precision medicine, we introduce a patient-specific definition of AHE in this study and propose deep learning based models to predict this novel definition of AHE in data from multiple independent institutions. We provide extensive evaluation of the models by studying their accuracies in detecting patient-specific AHEs with lead-times ranging from 10 min to 1 hour before the onset of the event. The resulting models achieve AUROC values ranging from 0.57–0.87 depending on the lead time of the prediction. We demonstrate the generalizability and robustness of our approach through the use of independent multi-institutional data.

## Introduction

Intensive care units (ICU) care for a hospital’s sickest patients who require around the clock monitoring and life saving treatment. In the ICU, complex patient conditions and ailments can develop suddenly and often manifest in physiologically unique ways. Despite the heterogeneity among the patient population, conventional ICU treatments generally adopt a “one-size-fits-all” approach, leading to variable patient outcomes and potentially ineffective use of hospital resources. As such, the concepts of precision medicine and individualized therapy have garnered increasing attention in critical care research^1^. To advance the concept of precision medicine, the development of effective and useful big data and machine learning applications has been of great interest^2–5^. Predictive modeling has often been explored in ICU research, and increasingly relies on machine learning methods. The motivation of such research is to predict future events that may be indicative of a deteriorating patient state so that preemptive measures may be taken to mitigate its potential impact. In particular, the prediction of the onset of an acute hypotensive episode (AHE) has been of interest to the critical care research community^6^. Hypotension is a term that denotes low blood pressure. An AHE is broadly defined as a sudden drop in blood pressure that is sustained over a period of time. Prolonged hypotension has been shown to be a risk factor for organ failure and death in the ICU^7,8^. Advanced warning of a AHE can prompt the clinical team to investigate developing illness states such as occult bleeding or sepsis.

The prediction of AHE using computational methods was spurred by the annual Computing in Cardiology/Physionet Challenge in 2009, in which participants were tasked with predicting the onset of an AHE up to an hour in advance. The definition of AHE used in this event was specified as a period of 30 min or more in which 90% of mean arterial blood pressure (MAP) readings were below 60 mmHg^6^. This definition of AHE has been commonly used in the literature when developing predictive models for the ICU since the initial challenge^9–13^. However, as discussed by Moody et al.^6^, common false positive predictions of an AHE in many of these studies may have been annotated as an actual episode by a less restrictive definition and considered a true positive in practice. Aside from the physionet definition of AHE, a paper by Hatib et.al.^14^ proposed a definition of a period of 1 min or greater in which a patient’s MAP fell below 65 mmHg. Prior literature has explored a number of computational methods to analyze ICU data and forecast the rigid threshold definition of AHE. Many studies have used statistical features derived from a window of data prior to the forecast window. For example, the winner of the 2009 Physionet Challenge, Chen et al.^15^, utilized a simple rule-based classification scheme using indices derived from as little as 5 min of data prior to the forecast window. Recently, more sophisticated feature extraction techniques have been explored by Hatib et al.^14^, in which commercial software extracted features such as cardiac output, stroke volume, and slope from high-fidelity arterial waveforms to predict AHE through logistic regression.

The conventional definition of AHE and other similar constructs is based on a single cutoff value and thus may not be suitable for individual patients. Normal blood pressure varies between individuals and patients may tolerate hypotension to varying degrees before organ damage ensues. In treating critically ill patients, it has been suggested that different blood pressure targets may improve outcomes for certain subgroups of patients^16^. In patients unlikely to benefit from this, personalized management can mitigate the complications and side effects of treatments intended to raise blood pressure. It may also prompt treatment for patients whose blood pressure is relatively low for their physiologic needs, but which might not otherwise be flagged as abnormal. Therefore, the conventional threshold-based approach to describe an AHE fails to capture patient specific dynamics that can be encountered in the ICU.

In the current literature, state of the art techniques such as deep learning have not been applied to the prediction of hypotensive episodes. The ability of deep methods to handle sequences of large data and automatically engineer features is well suited for the use of multivariate physiological time series data collected in the ICU^17,18^. In particular, recurrent neural networks (RNNs) are of interest when analyzing sequential data. RNNs are characterized by the loop-like connections of nodes in the network, allowing the output of a prior step in learning to be used as input to the current step. This feedback allows such networks to retain memory of recent input events^19^. One of the limitations of the RNNs is difficulties in learning long-term dependencies^20^. To mitigate this, Long Short-Term Memory (LSTM) networks which use “information gates” to overcome the problem of vanishing gradients were proposed^19^. These gates allow LSTM modules to not only retain memory from relevant past data, but also forget temporal data that are unnecessary for the final prediction. Another flavor of RNN, the Gated Recurrent Unit (GRU)^21^ overcomes the limitations of classical RNNs in a similar fashion. A recent modification of the GRU, namely the GRU-D proposed by Che *et at.* is able to tolerate missing data in its inputs and has been shown to perform well on data collected in the ICU^22^.

Most prior literature on the prediction of AHE lack the use of an institutionally independent validation data set and thus raise questions regarding the ability of developed algorithms to generalize across care centers. Furthermore, generalization of predictive machine learning algorithms in medicine has been recently demonstrated to be difficult in regards to other target outcomes such as disease activity^23^. To reflect the wider trend of international clinical and research efforts, the primary aim of this study is to introduce and incorporate a patient-specific definition of AHE in the context of predictive modeling in the ICU. In pursuit of a generalizable model, data routinely collected by bedside monitors found in many ICUs is used to develop a predictive model. To validate the generalizability of the proposed patient-specific AHE, and of the predictive models presented in this study, we utilize an independent multi-institutional hold-out dataset. Given the moving target nature of the patient-specific outcome and the sequential properties of the data being used, a deep learning approach explored with LSTMs and GRU-Ds is presented. To evaluate the predictability of a patient-specific AHE, we investigate the performance of models trained to forecast the onset of an AHE from 10 min up to 60 min in advance.

**Figure 1:**
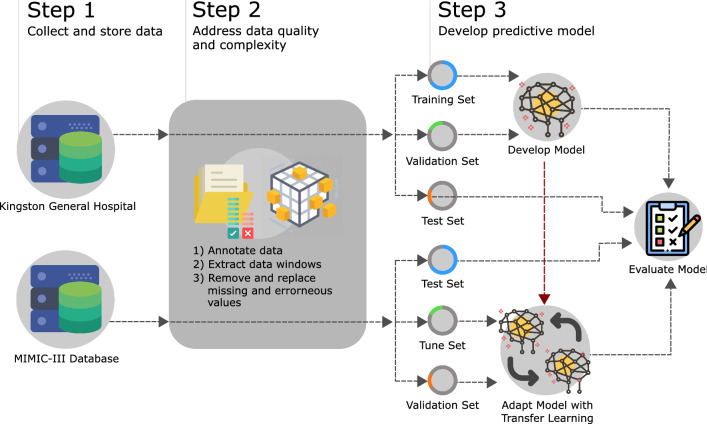
Experimental workflow and study design. Data from both the KGH and MIMIC-III databases are annotated and processed before being partitioned into three sets that are used for training, validation, and testing. Initial model development is performed using data from KGH exclusively. The models are then adapted via transfer learning using MIMIC-III data. All models are evaluated using held-out internal and external test sets.

**Figure 2:**
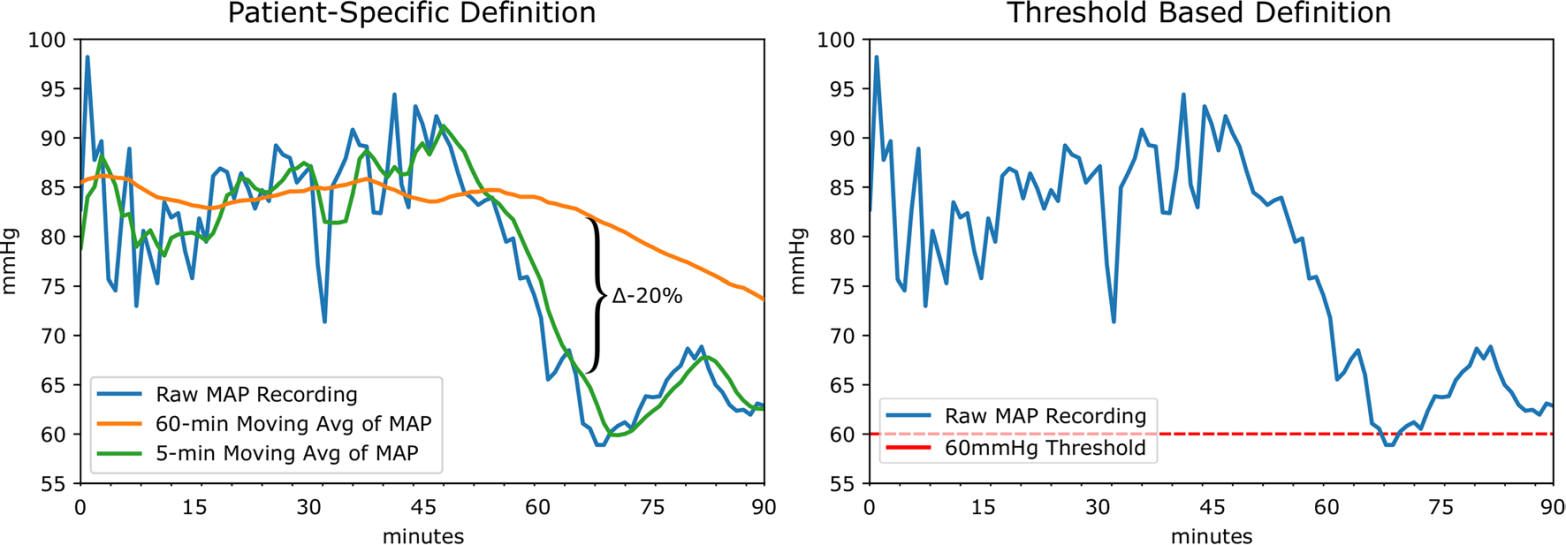
Comparing a patient specific definition (left) of AHE to the threshold based definition (right). Our patient specific definition is based on the use of two moving averages of MAP recordings in which the outcome of interest is a 20% drop in the averages. The conventional definition of AHE is based on a hard threshold such as 60 mmHg as highlighted in red. In this case, the threshold based definition would not annotate an AHE in this patient recording as a MAP below 60 mmHg is not sustained.

## Results

Two sources of data were used in this study: an internal cohort from Kingston General Hospital (KGH) and a cohort from the open-source MIMIC-III database. KGH data was used to develop the deep models in addition to logistic regression and Support Vector Machine (SVM) models for comparison. All models were evaluated on independent test sets from both the internal and external data sources. The experimental workflow is summarized in Fig. 1. Figure 2 demonstrates the concept of patient-specific definition of AHE. Comparing the patient specific definition (left) of AHE to the threshold based definition commonly used currently (right) is demonstrated in a sample patient recording in this figure. The patient specific definition we present is based on the use of two moving averages of MAP recordings in which the outcome of interest is a 20% drop in the averages. The conventional definition of AHE is based on a hard threshold such as 60 mmHg as highlighted in red. In this case, the threshold based definition would not annotate an AHE in this patient recording while the drop demonstrated around the 60 min recording time would be a clinically significant event to be aware of.

In this study, the prediction of the onset of an AHE is explored with respect to the amount of data needed to make a prediction and the amount of time the prediction can be made in advance. The data utilized by the model to make a prediction is referred to as an observation window throughout this paper, whereas the time a prediction is made in advance of the onset of an AHE is referred to as the gap length.

**Figure 3:**
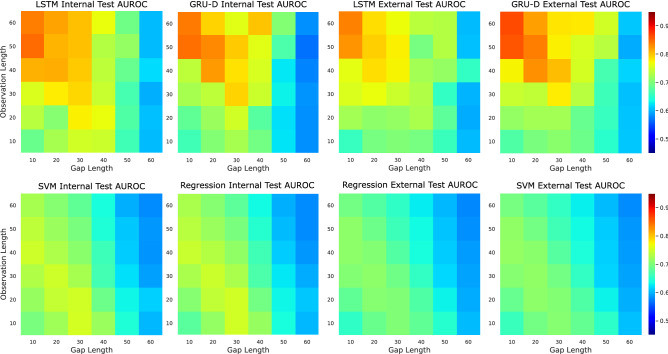
Comparison of AUROC between the logistic regression models, SVM models, and deep models on both the internal and external test sets at each investigated observation and gap length.

Figure 3 shows the achieved area under the receiver operator characteristics curve (AUROC) for our proposed deep learning solutions in the form of a colormap matrix. We also compare the results with a logistic regression and SVM approach in this figure. Each matrix illustrates the respective AUROC of models at each explored observation length and gap length. The four matrices on the right side of Fig. 3 shows the scores of the models evaluated on the external test set, while the four matrices on the left side show the scores of the models on the internal test set. For the deep learning models evaluated on the internal test set, the range of AUROC scores is [0.60–0.85] and [0.57–0.85] for the LSTM and GRU-D models respectively. In comparison, the AUROC scores of the same models evaluated on the external test set have a range of [0.59–0.84] and [0.59–0.87]. The logistic regression models reported a range of AUROC scores of [0.58–0.75] on the internal test set and [0.58–0.71] on the external test set. The SVM models performed similar to the regression models, with AUROC ranges of [0.57–0.75] and [0.57–0.70] for the internal and external test sets respectively. There is a general trend of decreasing model performance as the predictive gap length increases in both the regression and deep models, and deep models outperform the logistic regression classifier at a majority of the gap lengths explored.

**Figure 4:**
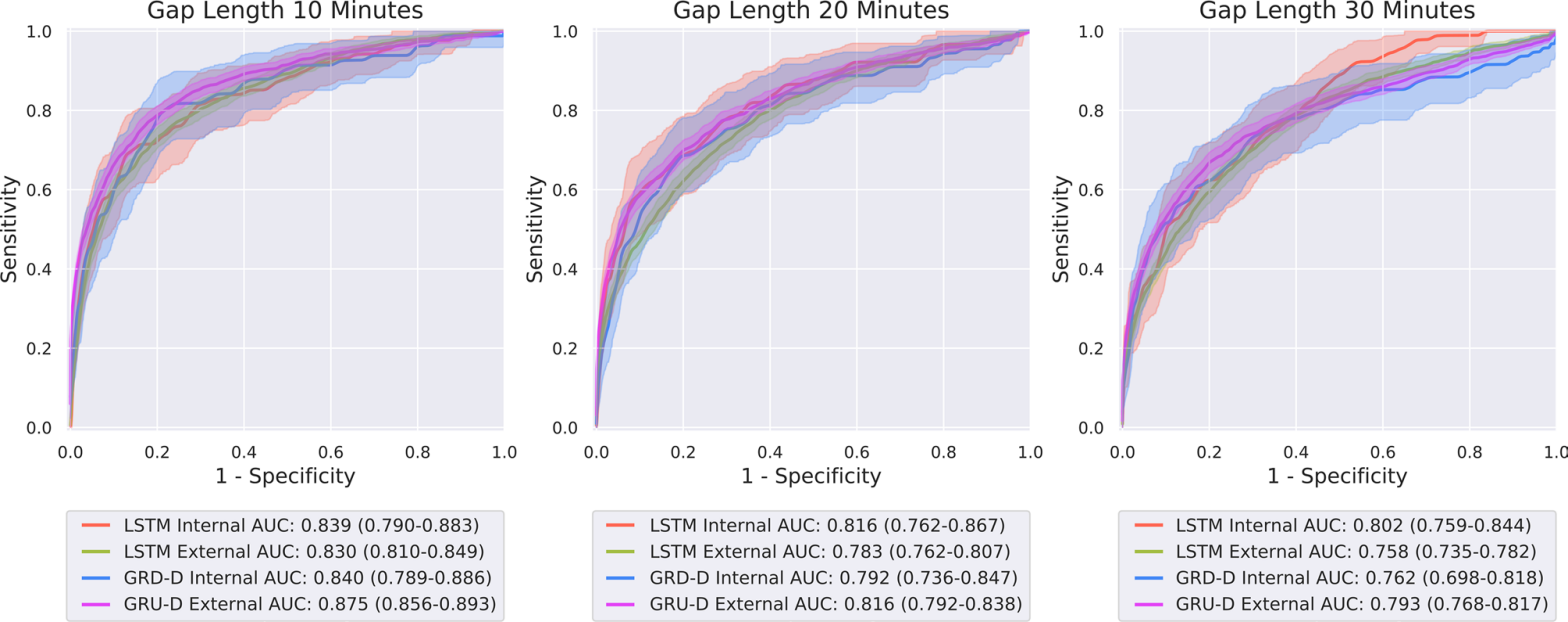
Comparison of ROC curves and corresponding 95% confidence intervals of models trained to make predictions using a 60 min observation window at a gap length of 10 (left), 20 (center), and 30 (right) minutes. Red curves denote performance of the LSTM model on the internal test set, green the LSTM model on the external test set, blue the GRU-D model on the internal test set, and purple the GRU-D model on the external test set.

Figure 4 depicts the receiver operator characteristic (ROC) curves and 95% confidence intervals of the deep models predicting at a gap length of 10-min, 20-min, and 30-min.

### Application of transfer learning

Across all explored combinations of observation and gap length the effects of transfer learning on AUROC scores when compared to the initial evaluation of the external test set ranged from [− 0.002 to + 0.01]. The highest gains in AUROC were observed when transfer learning was applied on the models trained to predict at a gap length of 40 min (+ 0.01). The largest decrease in AUROC was observed when transfer learning was applied to the model trained to predict at a gap length of 10 min (− 0.002).

## Discussion

In this study, we presented a generalizable model for the prediction of a patient specific definition of AHE that was externally validated on an institutionally independent dataset. Using RNN-based architectures, the developed models demonstrate generalizable results on data collected at an outside institution a decade before the collection of data from the local cohort. In almost all definitions of the prediction task, the deep models were able to outperform a logistic regression model on both the KGH and MIMIC-III test sets. The performance gains of the deep model are more noticeable when comparing against the external test set, suggesting the ability of the deep model to learn generalizable features that apply across institutions. Performance on the internal test set was observed to be marginally higher than the external test set in most definitions of the prediction task.

As seen in Fig. 3 there is a trend of increasing model performance when more historical physiological information is utilized by the model (larger observation window) and the time-to-prediction is reduced (smaller gap length). It is interesting to note that regardless of model type or use of internal or external test sets, there is a drop in performance around a gap length of 40–50 min.

The models did not demonstrate an increase in prediction performance when transfer learning was applied. A possible explanation may be that the amount of data used to fine tune the models during transfer learning was insufficient. The limited number of patients in the tune set may not offer an adequate representation of the target domain and as such may not have offered much to learn or adapt to. When further analysis was conducted to compare the distributions of the three vital signs of interest between internal and external test sets, it was found that they were relatively similar. Additionally, preliminary experiments involving training the deep models on the MIMIC-III dataset and testing on the KGH cohort did not demonstrate any clear performance gains. The similarities in distributions and model performance may support the notion that the domains are similar enough that transfer learning would have minimal effect. Conversely, transfer learning may not have benefited the performance of the proposed methodology as both the patient specific definition of an event and the deep models we built are inherently generalizable. This is particularly interesting to note as the bedside monitors used to collect sensor data in each cohort are of different make and model. Furthermore, it is noted that most equipment manufacturers of patient monitors have some form of artifact removal process integrated into the product^24^.

Our study has a number of key strengths. First, we introduced a patient-specific definition of AHE which accounts for individual physiologic responses among patients, and has improved face validity over conventional single-threshold definitions. Under prior definitions, such as where AHE are defined as having 90% of values below a MAP of 60 over a 30 min interval^6^, a patient with baseline MAP of 61 that drifted down to 59 for 30 min would be relegated to the same class as a patient with a baseline MAP of 90 that dropped precipitously to 40 over the same interval; these patients are clearly physiologically distinct. Our relative AHE definition overcomes these effects, providing a physiologically grounded and interpretable construct. Second, we validated our model in an entirely external dataset that was both temporally and geographically distinct from the first. This practice, though essential for the development of generalizable prediction models, is not often seen in clinical machine learning studies.

Our study has some limitations as well. First, our AUROC values did not exceed 0.9, suggesting discrimination that was good but not excellent. When analyzing the performance of the models on a per-patient basis, it can be seen that a large number of the classification errors are contributed only by a handful of patients in both the internal and external test sets. In particular, a large number of false-negatives can be traced back to a subgroup of patients. Preliminary investigation into these patients done by examining the distribution of the recorded signals does not indicate any difference from the general cohort. It would be ideal to further investigate the characteristics of such patients in order to identify any systematic differences that might explain why classification performance was different in this group.

Second, we note that the positive predictive values (PPV) of the model were relatively low, ranging from 0.04 to 0.16 in the internal test set, and 0.07 to 0.32 in the external test set. This reflects the low prevalence of AHEs in the datasets ( 3%), which influences PPV, and has implications for clinical implementation. A decision support system based on this model would have to account for the low PPV in determining how to deal with positive alerts, specifically by implementing a response system that accounts for the likelihood of false alarms. With the current performance, a system that identifies low risk patients may perform better than one that identifies those at high risk. Our results suggest that AHE detection and prediction may benefit from specific measures to handle class imbalance in machine learning, which could lead to a higher PPV, and reduced rate of false alarms.

Lastly, in this study, we did not incorporate supplementary patient data such as diagnostic codes or notes, therefore this investigation is a topic of future research. Examination of patient-specific model performance also illustrates some of the strengths of the developed models and reported results. 414 of the 996 patients in the external test set had a classification rate of 90% or higher when considering a gap length of 10 min. At a classification rate threshold of 85%, 493 patients were covered.

Although the presented deep models perform similarly, it is discussed by Che et al. that the GRU-D model may be considered more interpretable^22^. A common drawback of deep learning methods can be their relative opacity. However, recent guidelines for the development of predictive models in a critical care setting emphasize the utility in making an accurate prediction so long as it is understood that the model makes no claim about causation^25^. Regardless, interpretability is an area of active interest in the research community and may be considered in future work.

The proposed definition of a hypotensive episode was defined to be a 20% drop in MAP. Although a larger drop may be clinically more significant, a preliminary annotation of the data indicated too few cases of larger drops in MAP to be considered feasible for study. Nonetheless, the ability of the developed models to predict a relatively subtle drop in MAP is notable. Further research is warranted to determine the optimal definition and its clinical significance when compared with the conventional definition of AHE.

In summary, this study demonstrates that the proposed patient-specific definition of a sudden episode of hypotension is generalizable across patient populations, and that the deep models used to predict these show generalizability between institutionally independent patient cohorts.

## Methods

### Data

The institutional dataset for model development was composed of data collected from the 33-bed combined medical and surgical tertiary care ICU retrospectively collected at the KGH in Canada between 2015 and 2019. The collection and use of the data in this study was granted ethical clearance by the institutional health ethics board. This study used archival data that was fully de-identified, and collected as part of routine clinical care. The local ethics board therefore waived the need to obtain informed consent. All methods were also developed in accordance with relevant guidelines and regulations. From the available data, 538 patients were identified to have a minimum of 2 hours of MAP recordings. Other than the minimum length requirement of MAP records, no further exclusion criteria were applied to patient selection. Exclusion criteria were intentionally chosen to be liberal to best approximate general performance metrics in an ICU.

The MIMIC-III database, composed of data collected from the Beth Israel Deaconess Medical Center in the United States between 2001 and 2012, was used for external validation^26^. A random subset of 1060 patients that adhered to the same exclusion criteria used in the KGH cohort was used in this study. Three physiological time-series were extracted as input features: MAP, Heart Rate (HR), and Peripheral Blood Oxygenation (SPO2%). To match the sampling frequency of the MIMIC-III vital signs data, the vital signs recordings of the institutional cohort was down-sampled from 0.5 Hz to a per-minute frequency using the mean value of the data points encompassing each 1 min window.

### Patient specific definition of AHE and annotation

**Figure 5:**
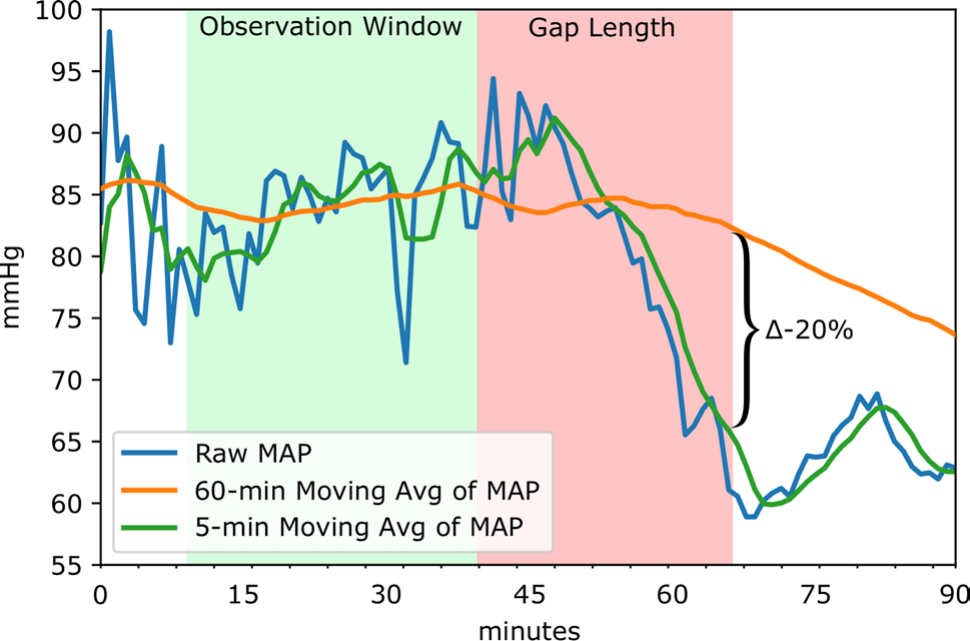
An example of an identified event in a patient recording. The annotated 20% drop is representative of the difference between a 60 min moving average (orange) and a 5 min moving average (green) of MAP. The relationship between the intervals of time that represent the observation window (highlighted green) and gap length (highlighted red) are also depicted relative to the identified drop in MAP.

The patient specific definition of an AHE used in this study was set out as a 20% or greater drop between a 60 min moving average and a 5 min moving average of a patient’s MAP recordings (Fig. 5). To be stringent against sensor drop-offs or abnormalities in the data, the MAP values 10 min after the initial detection of a 20% drop are required to be above 20 mmHg and not a “missing value”. Furthermore, it has been discussed that in patients undergoing surgery, a short period of time such as 10 min or less with a low MAP may increase the risk of kidney or heart injury^27^. As such, the minimum duration of an AHE with this proposed definition is 10 min.

To annotate the data, a sliding window approach was utilized. Each patient record was scanned sequentially, identifying points in the recordings in which a 20% drop in MAP was accompanied by the annotation criteria described above (10 min window of continuous MAP over 20 mmHg). The end of an AHE was also annotated as the point in time in which the difference between the 5 min and 60 min moving averages was less than 20%.

### Identification and extraction of data windows

In the development of a predictive model for the onset of an AHE, the data needed to make a prediction (observation window) and time-to-event (gap length) need to be considered when processing the data. Figure 5 depicts an arbitrary relationship between the observation window, gap length and the annotated drop in MAP. For the purpose of model development, a number of observation windows with associated class labels need to be extracted. When considering the binary prediction task of whether an AHE will occur or not, both positive and negative cases need to be defined.

A positive data sample is defined as an observation window in which an AHE onsets after a defined amount of time (gap length) from the end of the observation window. In the case of multiple AHEs occurring in the same patient recording, a minimum 2 hour gap was required between the end of an AHE and the beginning of an observation window of a following AHE. This 2 hour boundary was utilized to enforce a degree of independence of the following observation window. A negative data sample was defined as an observation window in which an AHE does not occur within 2 hour prior to the observation and within 2 hour after the end of the observation. In the case of multiple negative observation windows in a single patient case, none of the extracted samples overlapped one another. Negative data samples were also extracted from patient recordings that did not have any occurrence of an AHE during their stay. For each extracted window, the MAP, HR, and SPO2% recordings were extracted. The window extraction process was repeated for each of the studied gap lengths.

### Prepossessing

To inhibit the propagation or “leaking” of information beyond the bounds of the observation window, prepossessing is performed on the extracted windows of data rather than the entire patient record. For each identified and isolated observation window, both positive and negative labeled samples were processed to address missing and erroneous data points. First, values outside normal physiological ranges were temporarily removed and replaced with NaN. Second, missing values were replaced with backfilling—a method that propagates the next finite value backwards to replace missing values. To address the possibility of missing values at the end of the observation window, and to prevent the propagation of information of the future into the window, forwardfilling was used—a method that propagates finite values forward to replace missing values. As a means of quality assurance, windows with more than 10% erroneous values were excluded from use in this study. Erroneous values in this case are defined as a combination of both values outside normal physiological ranges and the occurrence of NaN values. For the experimentation with the missing value tolerant GRU-D architecture, a separate instance of the data was processed without backfilling or forwardfilling applied.

To improve the training of the Deep Models, the data were normalized to a [0, 1] scale for each feature using a per-feature static range. The static ranges for each feature are between 40 and 160 for both MAP and HR, and between 60 and 100 for SPO2%. These ranges were determined by consulting with physicians and analyzing the distribution of each data type across patients in the training cohort.

### Model development and evaluation

#### Division of data for model development

To properly gauge the performance of the model being developed, the available data was partitioned, by patient, into independent training, validation, and testing sets. Furthermore, to ensure independence of the sets, each data set was divided by patient rather than shuffling and dividing the extracted data windows.

The data from KGH was divided into a training set consisting of 380 patients, a validation set of 61 patients, and a test set of 74 patients. The training set contained 703 positive data samples and 18,643 negative data samples. The validation set contained 113 positive data samples and 2,933 negative data samples. The test set contained 114 positive data samples and 4,250 negative data samples. To address the severe class imbalance, the number of negative samples in both the training and validation sets were randomly down sampled to the number of positive samples in the respective set. Experiments with a training set composition of up to 66% negative cases and 33% positive cases were conducted, however, an evenly balanced training set was found to provide the best results. To provide a more robust measure of model performance in a real-world application, the test set retained all the available negative data samples.

The MIMIC-III data was randomly divided into tuning and validation sets for the purpose of domain adaptation using transfer learning and an independent test set that is used to evaluate the performance of each experiment conducted. The MIMIC-III cohort consisted of a total of 1060 patients. Data from 996, 29, and 35 patients were allocated to the test, tuning, and validation sets respectively. The test set contained 1,741 positive data samples and 31,237 negative data samples. The tune set contained 52 positive data samples and 574 negative data samples. The validation set contained 40 positive data samples and 808 negative data samples. Like the previously described KGH cohort, the number of negative samples in the tuning and validation sets were down sampled to match the number of positive cases. Similarly, the test set retained all negative data samples to evaluate the model on the full suite of data.

All models were evaluated on both the held out, independent KGH and MIMIC-III test sets. In addition to AUROC, sensitivity, and specificity as performance metrics, model performance was further evaluated by calculating confidence intervals through the use of bootstrapping^28^.

#### Comparative machine learning models

A logistic regression model was considered for initial experimentation, as the method is an approach commonly used for prediction tasks in critical care^14,29–31^. To establish a comparative baseline to existing literature, the mean of MAP over a given observation window is calculated as a feature to predict the onset of a hypotensive episode with a logistic regression classifier^15^. Additionally, an SVM model was considered for additional experimentation using the mean of MAP, HR, and SPO2% over an observation window as inputs. The regression and SVM models were fitted using the training set from the KGH cohort and evaluated on both the KGH and MIMIC-III test sets. For this study, six observation window lengths and six gap lengths were considered, each spanning a range of 10 min to 1 hour at 10 min increments. A model was fitted for each defined pair of observation window and gap length explored in this study, resulting in a total of 36 models being evaluated. Implementation of the models described was done in Python using the Scikit-learn package^32^. The development of the logistic regression models was conducted on a system with an Intel Core i7-7770k processor and 32GB of memory.

#### Deep models

**Figure 6:**
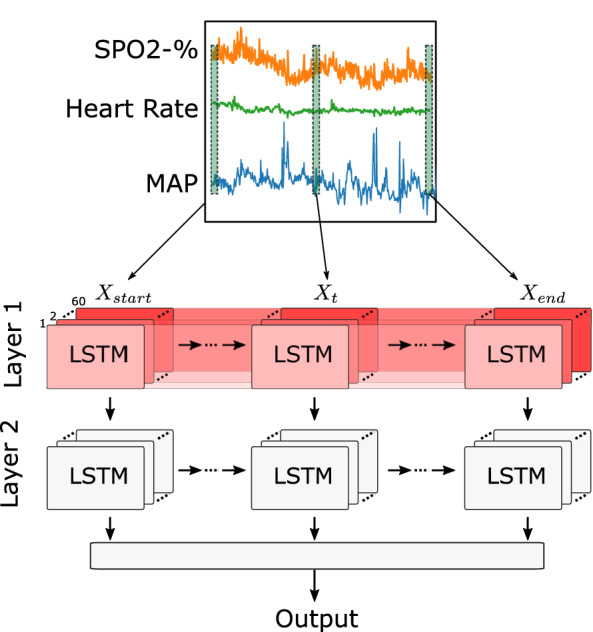
The architecture of the LSTM network. A window of physiological data containing MAP, HR, and SPO2% recordings is processed through 2 LSTM layers with the output of the last LSTM layer being merged in a final layer to make a binary prediction. LSTM units in the first layer are shown with different colors (unit 1: lighter red through unit 60: darker red).

To predict the onset of an AHE, we utilized LSTM networks as our main architecture for modeling. Through a coarse exploration and grid search of the number of LSTM layers and units, it was found that an architecture with two LSTM layers each with 60 units performed well on the data. The output of the second LSTM layer is fed into a fully-connected layer to predict a binary outcome representing the probability of an AHE occurring at a certain time into the future. As a result, our model has an input shape of [*number of time steps*, 3] and an output shape of 1. The number of time steps pertains to the number of observations per window, depending on the defined observation window length, this takes a value of 10, 20, 30, 40, 50, 60. The final dimension refers to the three input features, MAP, HR, and SPO2%. The structure of the network is summarized in Fig. 6.

Training is performed by minimizing a binary cross-entropy loss via stochastic gradient descent. The use of a cosine annealing schedule for learning rate has been shown to improve training in deep networks^33^. We used such an approach with warm restarts and the Adam optimization algorithm^34^ to update model parameters. Furthermore, as L2 regularization and dropout have been shown to effectively prevent overfitting of models to training data, we used an L2 regularization of 1e−3 for all parameters and a dropout of 0.4 between the LSTM and fully connected layers^35,36^ The value of the hyperparameters outlined above were determined using a grid search.

We also evaluated a GRU-D^22^ on the non-backfilled data. This architecture augments the base GRU architecture with a trainable decay to handle missing or irregularly-sampled data. In lieu of backfilling, the model is provided with three inputs: the original unfilled signal, an index identifying the timestamp of each observation and a mask on timesteps with invalid or missing data. In keeping with the training procedure documented by Che et al., all models used a fixed learning rate of 1e−4, dropout of 0.3 and a single recurrent layer. To compensate for lack of stacking, the recurrent unit count was increased from 60 to 100. Hidden layer size, loss and other hyperparameters matched those of the LSTM.

For each the LSTM and GRU-D architectures, a separate model was trained using the same hyperparameters for each combination of the observation window and gap length explored in this study. As a result, a total of 72 deep models were trained and evaluated (36 LSTM and 36 GRU-D models).

Models were implemented using tensorflow^37^ and took between 11 and 37 min to train with an average time of 13 min. The development of the models described were conducted on a system with an Intel Core i7-7700K CPU, 32GB of memory, and an Nvidia RTX 2070.

#### Application of transfer learning

To adapt the models to the target domain, model weights from the previously trained models were transferred to an identical model architecture and fine-tuned using a variable number of training samples from the tuning set, using the MIMIC-III validation set to select the best tuned model. The number of tuning samples used started at 10 and increased by 10 to a maximum of 50. The set of samples used to tune the network were class balanced to an equal number of positive and negative samples. The fine tuning process utilized the same optimization and regularization techniques as the development of the initial models previously described, but with a reduced learning rate of 0.0001. A reduction in learning rate was adopted as the propose of the transfer learning was to fine-tune the models to the target domain rather than retrain the model.
